# Automatic recognition of parasitic products in stool examination using object detection approach

**DOI:** 10.7717/peerj-cs.1065

**Published:** 2022-08-17

**Authors:** Kaung Myat Naing, Siridech Boonsang, Santhad Chuwongin, Veerayuth Kittichai, Teerawat Tongloy, Samrerng Prommongkol, Paron Dekumyoy, Dorn Watthanakulpanich

**Affiliations:** 1 Center of Industrial Robot and Automation (CiRA), College of Advanced Manufacturing Innovation, King Mongkut’s Institute of Technology Ladkrabang, Bangkok, Thailand; 2 Department of Electrical Engineering, School of Engineering, King Mongkut’s Institute of Technology Ladkrabang, Bangkok, Thailand; 3 Faculty of Medicine, King Mongkut’s Institute of Technology Ladkrabang, Bangkok, Thailand; 4 Mahidol Bangkok School of Tropical Medicine, Faculty of Tropical Medicine, Mahidol University, Bangkok, Thailand; 5 Department of Helminthology, Faculty of Tropical Medicine, Mahidol University, Bangkok, Thailand

**Keywords:** Object detection approach, Parasitic products, YOLO, Parasite image dataset, Parasite products recognition

## Abstract

**Background:**

Object detection is a new artificial intelligence approach to morphological recognition and labeling parasitic pathogens. Due to the lack of equipment and trained personnel, artificial intelligence innovation for searching various parasitic products in stool examination will enable patients in remote areas of undeveloped countries to access diagnostic services. Because object detection is a developing approach that has been tested for its effectiveness in detecting intestinal parasitic objects such as protozoan cysts and helminthic eggs, it is suitable for use in rural areas where many factors supporting laboratory testing are still lacking. Based on the literatures, the YOLOv4-Tiny produces faster results and uses less memory with the support of low-end GPU devices. In comparison to the YOLOv3 and YOLOv3-Tiny models, this study aimed to propose an automated object detection approach, specifically the YOLOv4-Tiny model, for automatic recognition of intestinal parasitic products in stools.

**Methods:**

To identify protozoan cysts and helminthic eggs in human feces, the three YOLO approaches; YOLOv4-Tiny, YOLOv3, and YOLOv3-Tiny, were trained to recognize 34 intestinal parasitic classes using training of image dataset. Feces were processed using a modified direct smear method adapted from the simple direct smear and the modified Kato-Katz methods. The image dataset was collected from intestinal parasitic objects discovered during stool examination and the three YOLO models were trained to recognize the image datasets.

**Results:**

The non-maximum suppression technique and the threshold level were used to analyze the test dataset, yielding results of 96.25% precision and 95.08% sensitivity for YOLOv4-Tiny. Additionally, the YOLOv4-Tiny model had the best AUPRC performance of the three YOLO models, with a score of 0.963.

**Conclusion:**

This study, to our knowledge, was the first to detect protozoan cysts and helminthic eggs in the 34 classes of intestinal parasitic objects in human stools.

## Introduction

### Motivation

Intestinal parasitic diseases are the most common infectious disease in humans worldwide, with a higher prevalence in undeveloped countries, where people are isolated from civilization and live in unhygienic conditions, and they continue to be major public health issues in high-risk occupations and risk groups. Human intestinal parasites can be divided into the following two groups: helminths and protozoa, which are the causative pathogens of the diseases. In both children and adults, the risk of infection with intestinal parasites is relatively high (Mori et al., 2013). It has an impact on both physical and mental development as well as work efficiency and education, and this may have an impact on future population quality and the country’s long-term development (Prasertbun et al., 2019; Watthanakulpanich et al., 2011; Watthanakulpanich et al., 2013). As a result, efforts and expenses are paid to reduce parasitic infection because many other cofactors are involved (Anantaphruti et al., 2008). However, if a regular health check with stool examination is conducted at least once a year, the rate of parasitic infection in Thailand is likely to be decreased and better controlled, regardless of whether the urban communities hygiene issues and poor environmental sanitation or the rural communities lack toilets and water. As a result, artificial intelligence innovation for searching various parasitic products in stool examination will enable patients in remote areas to access diagnostic services. Subsequently, proper management with effective treatment can be provided for patients in order to improve the quality of life, especially to prevent complications and to decrease the death rate in remote areas far from the hospital services. Most intestinal parasites can be detected with a simple stool examination; however, due to the lack of equipment and trained personnel, basic stool examination is not always available to all people in remote areas. To correctly identify parasites, determine their true prevalent incidence, and provide prompt treatment, an effective tool is required.

Many studies have been conducted to detect and classify medically significant parasites using microscopic image analysis. Several studies in animals successfully classified several parasites in pigs and cattle with morphologic feature extractions using interactive image analysis tools (Daugschies, Imarom & Bollwahn, 1999; Joachim, Dülmer & Daugschies, 1999; Sommer, 1998). Vision-based detection and the classification method were used to detect *Trichuris suis* eggs using match filters for recognition; longitudinal anisotropy, and mean scattering intensity for biological feature extraction; and linear-quadratic discriminant analysis for classification (Bruun, Kapel & Carstensen, 2012). Several parasitic eggs in pigs and cattle were separated and classified using morphological feature numerical extractions (Daugschies, Imarom & Bollwahn, 1999; Sommer, 1996). A low-cost, automated parasite-diagnostic system based on a trained U-Net convolutional neural network (CNN) model was proposed to receive both egg species and egg counts for six different kinds of animal intestinal parasites (Li et al., 2019). In humans, a computer processing algorithm was used to classify seven different types of human helminthic eggs (Yang et al., 2001). A robust technique based on invariant moments (IM)—an adaptive network based fuzzy inference system (ANFIS) was proposed for feature extraction and recognition to classify 16 different intestinal parasitic eggs (Dogantekin et al., 2008). Furthermore, a method based on IM and multiclass classification using support vector machine was investigated to classify 16 protozoa and helminth objects (Avci & Varol, 2009). The robust method, which used phase coherence technology for image segmentation and the support vector machine method for the classification of six different parasitic eggs, yielded satisfactory results with a 95% recognition rate (Li et al., 2015). Deep learning with CNN models has recently conquered various research studies of medical imaging applications with recognition of intestinal parasites (Zhou et al., 2021). Furthermore, the FecalNet, which is based on a combination of deep learning and multiple neural networks, was proposed for automatic detection and identification of six common fecal components (Li et al., 2020b). In addition, there are two well-known categories in object detection approach: region proposal based two-stage detector and regression/classification based one-stage detector (Zhao et al., 2019). Indeed, the two-stage detector had high accuracy in localization and object recognition because it used region proposal network (RPN) for object bounding boxes and Region of Interest Pooling (RoIPool) for feature extraction in candidate bounding boxes classification and regression task. The one-stage detector, on the other hand, had a high inference speed because it directly predicted the bounding boxes without the RPN step (Jiao et al., 2019). For eight classes of human parasitic eggs in stool images, an automatic parasite-worm egg detection method based on the Faster R-CNN (regions with CNN features) two-stage detector was studied and reported 97.67% in mAP (Viet, ThanhTuyen & Hoang, 2019). This work motivated us to apply object detection model in the recognition of intestinal parasite eggs.

### Related works

More recently, the popular one-stage detection model, You Only Look Once (YOLO) became increasingly interesting and was being continuously developed in the field of object detection (Bochkovskiy, Wang & Liao, 2020; Redmon et al., 2016; Redmon & Farhadi, 2017; Redmon & Farhadi, 2018). YOLO detected multiple objects directly by predicting multiple bounding boxes and class probabilities. These YOLO algorithms and its modifications have successfully demonstrated on target recognition of objects in an image. With the subsequent improvement of accuracy and computational performances, YOLO based network architectures, such as YOLOv3 (Kuznetsova, Maleva & Soloviev, 2020; Lawal, 2021), YOLOv4 (Roy & Bhaduri, 2022; Roy, Bose & Bhaduri, 2022) and YOLOv5 (Fan et al., 2022; Gu et al., 2022), were applied in many research works. Although YOLOv5 is the latest version of YOLO, the comparisons between YOLO version have been still opened as question. In literature, YOLOv3 has higher precision and speed than another state-of-the-art algorithm namely Faster R-CNN (regional convolutional neural network) and SSD (single shot detector) (Li et al., 2020a; Zhao & Ren, 2019). YOLOv4 has higher accuracy and detection speed in comparison with YOLOv3 and YOLOv5 (Bochkovskiy, Wang & Liao, 2020; Long et al., 2020; Rahman et al., 2021). In addition, some research studies reported the three YOLO comparison. It was found that YOLOv5 is higher accuracy than YOLOv3 and YOLOv4 whereas the detection speed of YOLOv3 is faster compared to that of YOLOv4 and YOLOv5 (Ge et al., 2021; Nepal & Eslamiat, 2022). According to the reported results, the latest three YOLO algorithms have different advantages and disadvantages. Due to the lower accuracy of YOLOv3, larger weight of YOLOv4, and complex network structure of YOLOv5, a more simplified version of YOLOv4 (YOLOv4-Tiny) was designed to maximize detection speed and improve computational efficiency (Wang, Bochkovskiy & Liao, 2021). In particular, it has been applied to the pine wilt disease detection (Li et al., 2021), trash detection (Kulshreshtha et al., 2021), multi-object tracking (Wu et al., 2021), electronic component detection (Guo et al., 2021), construction machinery and material identification (Yao et al., 2022), and fruit flies gender classification (Genaev et al., 2022). Based on the literatures, our research also focused on the fourth tiny model of YOLO, namely the YOLOv4-Tiny because it produces faster detection results and uses less memory with the support of low-end GPU devices.

Because object detection is a developing approach that has been tested for its effectiveness in detecting intestinal parasitic objects such as protozoan cysts and helminthic eggs, it is suitable for use in remote areas of undeveloped countries where many factors supporting laboratory testing are still lacking. The prevalence of parasitic infection remains high, and there is a need to provide people in these areas with comprehensive basic stool examination services as well as prompt treatment. Furthermore, previous research that used both traditional object detection and deep learning approaches, detected and classified a maximum number of 20 classes of intestinal parasite eggs. This study was carried out, and the YOLOv4-Tiny model was proposed for automatic recognition of 34 common classes of protozoan cysts and helminthic eggs in parasitic products of stool examination. For model comparisons, we used the YOLOv3 and YOLOv3-Tiny models.

## Materials & Methods

**Figure 1:**
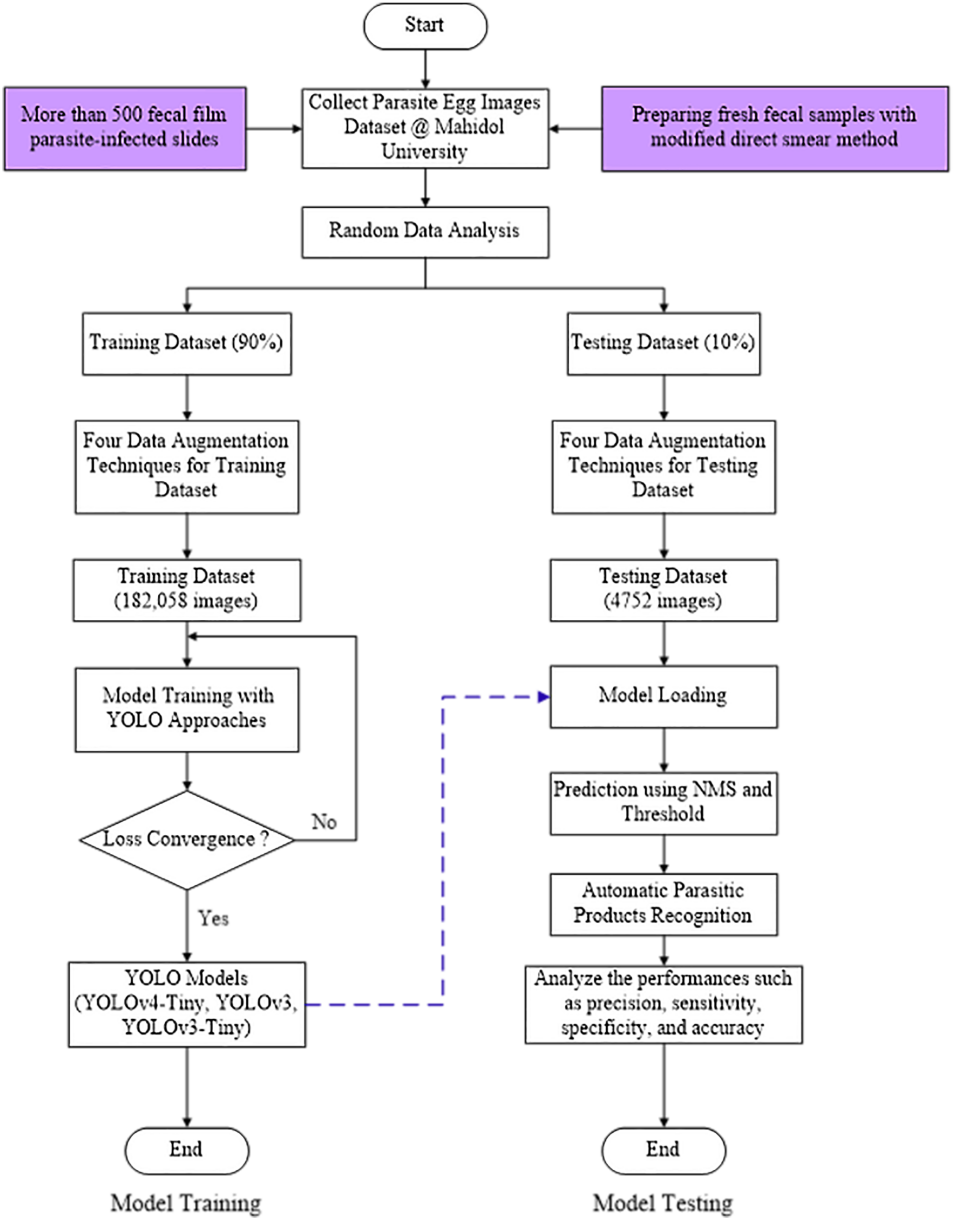
YOLO-based model flowchart for training and testing process of Parasite Products Recognition.

Figure 1 depicts the YOLO-based model flowchart for the Parasite Products Recognition training and testing process used in this study.

### Ethics statement

This study was approved by the Ethics Committees of the Faculty of Tropical Medicine, Mahidol University to anonymously use human left-over stool specimens collected in the Department of Helminthology, Faculty of Tropical Medicine, Mahidol University (Approved No. MUTM 2020-041-01). We strictly adhered to the university’s relevant guidelines and regulations.

### Fecal film preparation

The fecal film preparation method was modified from the simple direct smear and the modified Kato-Katz technique, which were widely used in general laboratories to diagnose common protozoan cysts and helminthic eggs. Each fecal sample was placed on absorbable paper, and the top of the sample was pressed with a wire net (105 mesh). The feces that had passed through the wire net were then withdrawn from the wire net and conveyed to the central hole of a disposal rectangular cardboard (3 × 4 × 0.137 cm) that was placed over a microscopic slide. The central hole had become clogged with feces. The cardboard was carefully removed, and the feces were left on the slide. After that, one drop of 0.85% normal saline solution and one drop of 1% Lugol’s solution were placed on each side of a slide, and half of the feces were added and stirred until the entire fecal sample was evenly suspended. A coverslip (22 × 30 cm) was gently placed on top of each fecal avoiding air bubbles. This procedure was performed at the Prayong Radomyos Laboratory, Mahidol Bangkok School of Tropical Medicine, Faculty of Tropical Medicine, Mahidol University. Finally, the fecal film preparation was ready for examination under a microscope using either low-power (×10) or high-power (×40) objective lens, and all intestinal parasitic objects were examined on both coverslips. If properly and skillfully examined, two modified direct smears were sufficient for the detection of protozoan cysts and helminthic eggs.

### Parasite image dataset

**Figure 2:**
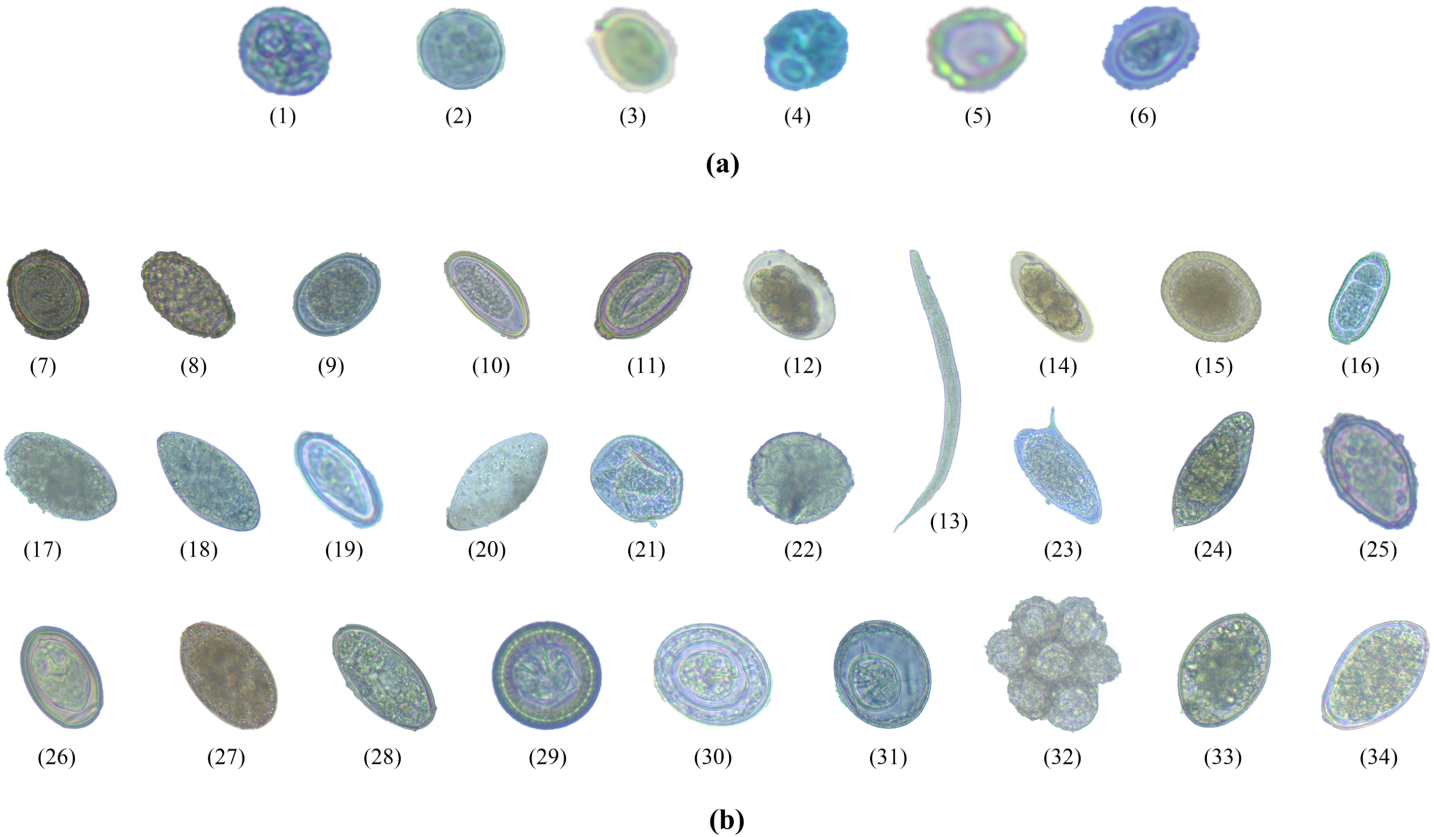
The total 34 classes of parasitic objects: (A) protozoan cysts and (B) helminthic eggs.

Image dataset was created by collecting a large number of images with specific features of each type of intestinal parasitic object from the fecal sample preparation. Trained laboratory examiners typically visually examine fecal specimens at magnifications ranging from 100- to 400-fold. The trained examiners identified protozoan cysts and helminthic eggs by inspecting the features of the parasitic objects such as the cyst-wall and egg-shell color, size, and shape. The required images were obtained from more than 500 fecal films of parasite-infected samples at Department of Helminthology, Faculty of Tropical Medicine, Mahidol University. Intestinal parasitic objects were photographed using an Axiocam Camera embedded in a ZEISS Primo Vert Microscope connected with Zen 2.3 (blue edition) software on a Windows 10 installed ACER desktop computer for the image datasets. All images were captured at a magnification of 40 ×, with dimensions of 2,560 × 1,920 pixels, 24-bit depth, and JPG file format. Figure 2 shows the six protozoan cyst classes and the 28 helminthic egg classes of intestinal objects in human stools.

The training and testing process was the most important factor influencing the success of deep learning (Uçar et al., 2020). The training-testing ratio used in the literature ranged from 50%–95% for training and 5%–50% for testing. In our study, the YOLO approaches were designed by 90%:10% for the training-testing dataset for total image training and image testing, respectively (Table 1). Using 1,597 images from a 90% dataset, the three YOLO models were trained to recognize different types of protozoan cysts and helminthic eggs. The trained YOLO models were put to the test with a variety of new images of intestinal parasitic objects, including protozoan cysts and helminthic eggs. They were tested for interpretive accuracy using 176 images from a 10% dataset.

**Table 1:** The random division of the image dataset for training and testing process.

Classes		Abbreviation (Abb.) in labeling images	Abb. in confusion matrix	No. of images	Training (90%)	Testing (10%)
**Protozoa**						
1	*Entamoeba histolytica*	*E. histolytica*	EHI	35	31	4
2	*Entamoeba coli*	*E. coli*	ECO	62	56	6
3	*Endolimax nana*	*E. nana*	ENA	76	68	8
4	*Iodamoeba butschlii*	*I. butschlii*	IBU	44	40	4
5	*Blastocystis* spp.	*Blastocystis* spp.	BLA	41	37	4
6	*Giardia duodenalis (Giardia intestinalis, Giardia lamblia)*	*G. duodenalis*	GDU	91	82	9
**Helminths**						
7	*Ascaris lumbricoides* fertilized egg	*A. lumbricoides* (Fertilized egg)	ALF	173	156	17
8	*Ascaris lumbricoides* unfertilized egg	*A. lumbricoides* (Unfertilized egg)	ALU	29	26	3
9	*Ascaris lumbricoides* decorticated egg	*A. lumbricoides* (Decorticated egg)	ALD	43	39	4
10	*Enterobius vermicularis*	*E. vermicularis*	EVR	33	30	3
11	*Trichuris trichiura*	*T. trichiura*	TRI	33	30	3
12	Hookworm	Hookworm	HOO	28	25	3
13	*Strongyloides stercoralis*	*S. stercoralis*	STE	44	40	4
14	*Trichostrongylus orientalis*	*T. orientalis*	TOR	18	16	2
15	*Toxocara* spp.	*Toxocara* spp.	TOX	71	64	7
16	*Capillaria philippinensis*	*C. philippinensis*	CPH	33	30	3
17	*Fasciolopsis buski*	*F. buski*	FBU	72	65	7
18	*Echinostoma* spp.	*Echinostoma* spp.	ECH	28	25	3
19	*Haplorchis* spp.	Haplorchis spp.	HAP	39	35	4
20	*Gastrodiscoides hominis*	*G. hominis*	GHO	45	40	5
21	*Schitosomia japonicum*	*S. japonicum*	SJA	24	22	2
22	*Schistosoma mekongi*	*S. mekongi*	SME	68	61	7
23	*Schistosoma mansoni*	*S. mansoni*	SMA	89	80	9
24	*Schistosoma haematobium*	*S. haematobium*	SHA	34	31	3
25	*Opisthorchis viverrini*	*O. viverrini*	OVI	50	45	5
26	*Eurytrema pancreaticum*	*E. pancreaticum*	EPA	26	23	3
27	*Fasciola* spp.	*Fasciola* spp.	FAS	31	28	3
28	*Paragonimus* spp.	*Paragonimus* spp.	PAR	121	109	12
29	*Taenia* spp.	*Taenia* spp.	TAE	53	48	5
30	*Hymenolepis nana*	*H. nana*	HNA	29	26	3
31	*Hymenolepis diminuta*	*H. diminuta*	HDI	53	48	5
32	*Dipylidium caninum*	*D. caninum*	DCA	62	56	6
33	*Diphyllobothrium latum*	*D. latum*	DLA	35	31	4
34	*Spirometra* spp.	*Spirometra* spp.	SPI	60	54	6
**Total**				1,773	1,597	176

After randomly dividing the image dataset for training and testing, image labeling was applied manually to the training dataset. Image labeling is an important step in model training because the model can learn from labeled image datasets. The model’s accuracy is also determined by the quality of the training dataset. Manual image labeling can identify the regions of an object in an image and create text-based descriptions of those regions for classification. In our study, image labeling was performed using the CiRA CORE platform (CiRA CORE Development Group, 2021) before image augmentation techniques were applied.

### Image augmentation

Generally, image augmentation was often used to expand the data in order to obtain sufficient information. The amount of picture data points was raised above that of the initial dataset to allow the in-house deep learning platform (CiRA CORE) to be trained to detect intestinal parasitic objects in feces with high accuracy. The following four image-transformation techniques were used in this study: rotating the original image (rotation), changing the lighting condition of the image (contrast), adjusting image clarity (blur), and adding Gaussian noise to the image (noise). As a result, one image was divided into multiple separate images in datasets, which were then merged with the original set of photos and used in the object detection method training practice with the three YOLO models. The image dataset for each parasite class was divided into two parts, as shown in Table 1: the training set, which comprised approximately 90% of the dataset, and the testing set, which comprised the remaining 10% of the dataset and included no duplicate photographs from the training set.

The augmentation method was used twice in this experimental study. The first time was prepared to create diverse images for the training dataset using the four data augmentation strategies listed below:

• Rotation: Each image was received by rotating 45° between −180° and 180°; and nine different scales were used.
• Contrast: Adjusted between the brightness and darkness areas of each image in 0.2 increments from 0.4 to 1.1 in pixel multiplication range, and four different scales were selected.
• Noise: Gaussian noise was injected into each image in increments of 10 from 0 to 30 in the standard deviation range, and four different scales were chosen.
• Blur: Each image was blurred with a Gaussian filter with a standard deviation of 9 and only one scale was used.

**Figure 3:**
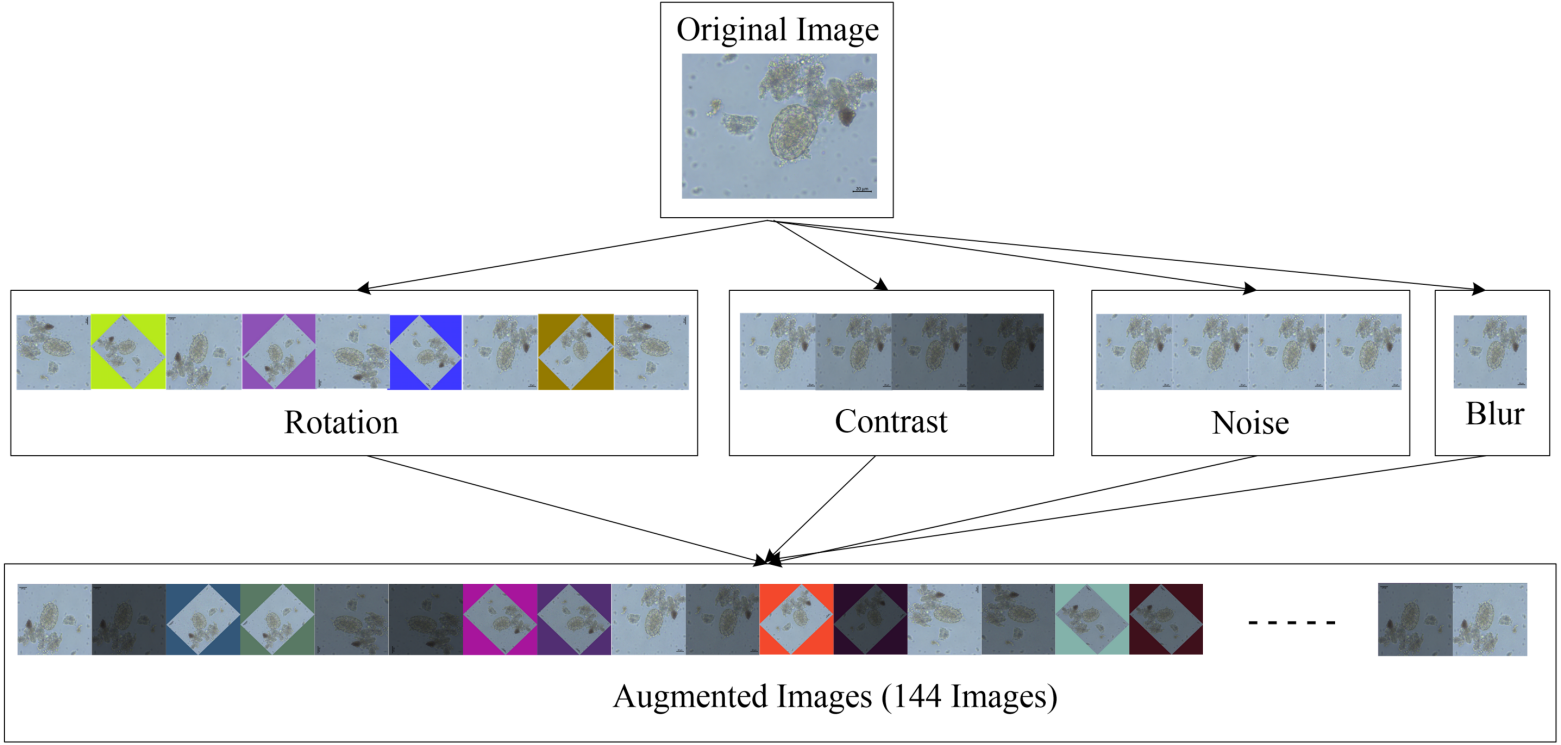
Image augmentation for training dataset.

When all four augmentation techniques are used together, a single original image can be expanded to 144 images. Because the training dataset contained 1,597 images, a total of 182,058 images with a resolution of 608 × 608 pixels were acquired. Figure 3 shows an example of an augmented image.

The second time, the testing dataset was prepared using the four data augmentation techniques listed below:

• Rotation: Each image was received by rotating 90° from 0° to 180°, and three different scales were chosen.
• Contrast: Adjusted between the brightness and darkness areas of each image in 0.2 increments from 0.4 to 1.1 in pixel multiplication range, with four different scales selected.
• Noise: Gaussian noise was injected into each image in increments of 15 from 0 to 30 in the standard deviation range, with three different scales chosen.
• Blur: Each image was blurred with a Gaussian filter with a standard deviation of 9 and only one scale was used.

**Figure 4:**
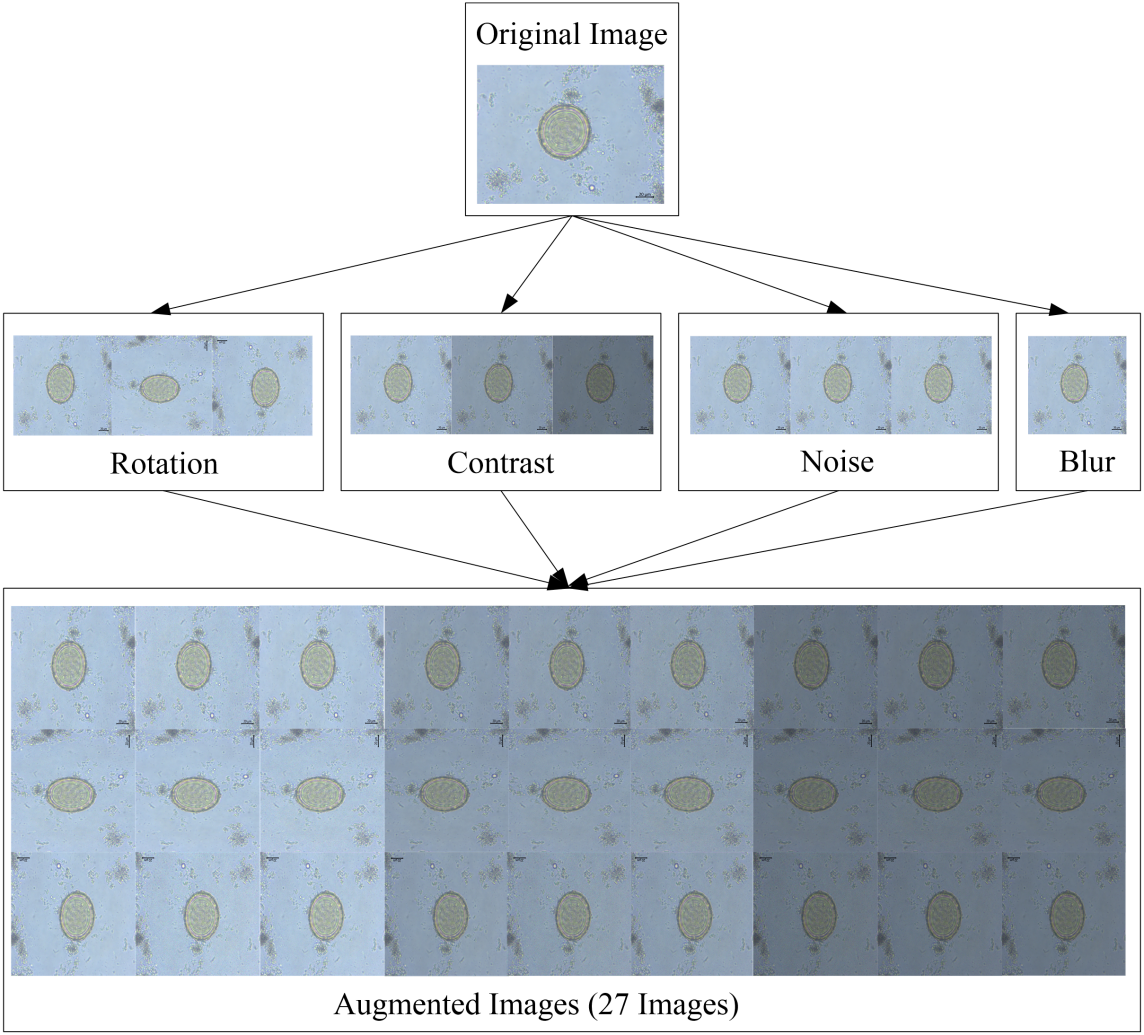
Image augmentation for testing dataset.

When these four augmentation techniques are used in tandem, one original image can be given rise to 27 images. Because the training dataset contained 176 images, the testing dataset contained a total of 4,752 images with a resolution of 608 × 608 pixels. Figure 4 depicts an example of an augmented image.

### Parasite egg detection with YOLOv4-Tiny object detection model

**Figure 5:**
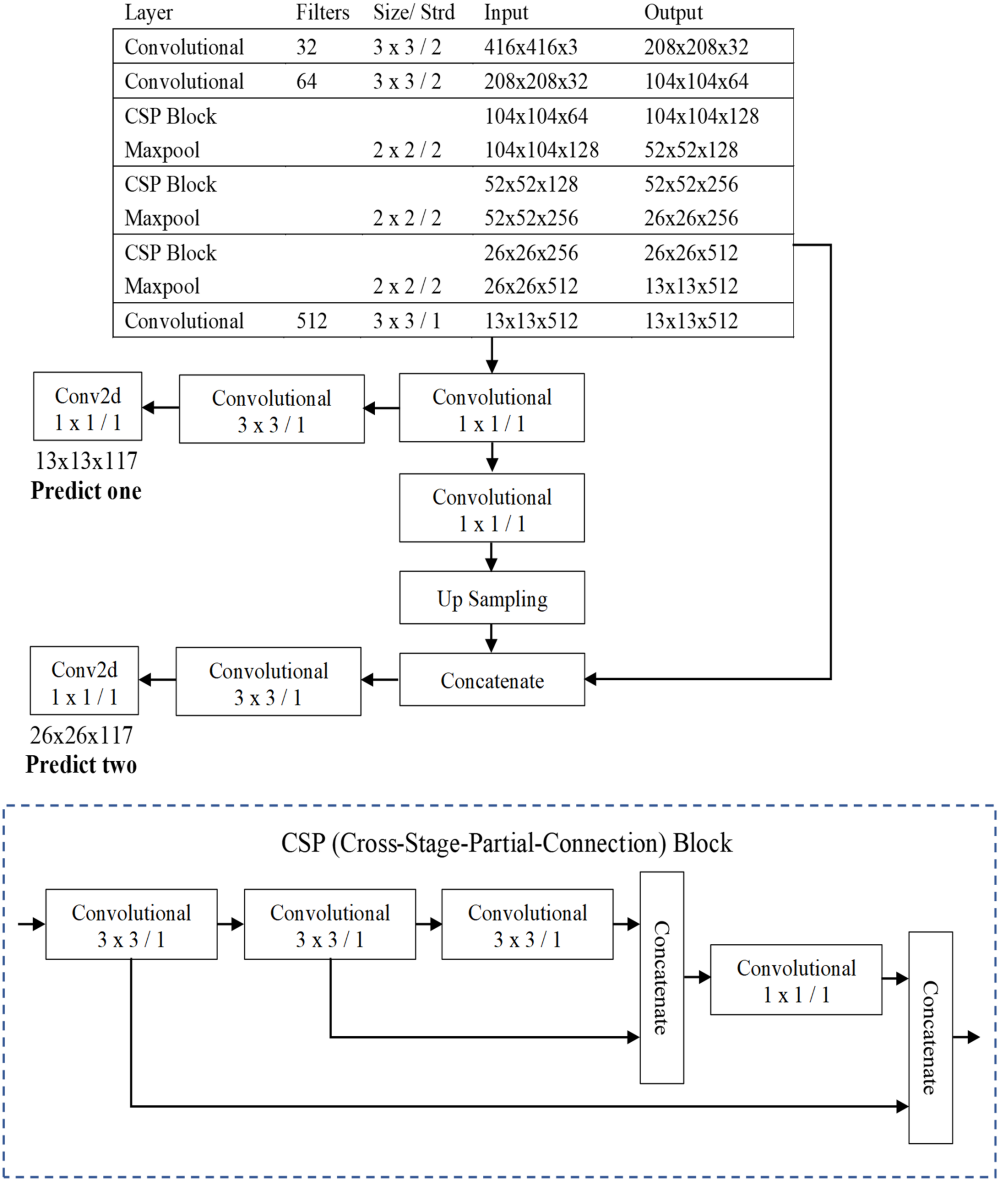
The architecture of the YOLOv4-Tiny model.

The three well-known CNN-based one-stage object detection models namely YOLO were used in this paper for the automatic recognition of protozoan cysts and helminthic eggs. This study primarily proposed a compressed version of YOLOv4 (YOLOv4-Tiny), which is intended for training on machines with limited computing power. The fourth version of YOLO proposed by Bochkovskiy, Wang & Liao (2020) improved processing speed and object detection accuracy. Unlike previous YOLO versions, YOLOv4 employed the CSPDarknet53 backbone as the feature extractor. In place of the Res block module, the CSPDarknet53 is made of a convolutional building block and a CSP block module. The CSP block module outperformed the Res block module in terms of improving the convolutional network’s learning ability. Because the YOLOv4-Tiny design is based on YOLOv4, the architecture used the CSPDarknet53-tiny backbone as the feature extractor, as shown in Fig. 5. CSPDarknet53-tiny was built with three convolutional building blocks and three CSP blocks. The CSP block of YOLOv4-Tiny, unlike YOLOv4, employs the Leaky ReLU (Rectified Linear Unit) activation function rather than the Mish activation function. Furthermore, YOLOv4-Tiny employs complete intersection over union loss and a loss function composed of the following three components: bounding box regression loss, confidence loss function, and classification loss function. Finally, the YOLOv4-Tiny generates two-branch YOLO outputs that require fewer anchor boxes for prediction.

As shown in Fig. 5, the image’s 416 × 416 pixels were chosen for the input layer, and two different scales of the feature map, 13 × 13 and 26 × 26, were used for the output YOLO layers in this model. The following hyperparameters were used in the YOLOv4-Tiny experimental setup: batch size of 64; 500,200 maximum batches; 16 subdivisions; momentum of 0.9; weight decay of 0.0005; activation function of Leaky ReLU for convolutional layer and linear for output layer; multistep learning rate policy was used with a base learning rate of 0.00261 and multiplied by a factor of 0.1 at a step value of 400,000 and 450,000, respectively.

**Figure 6:**
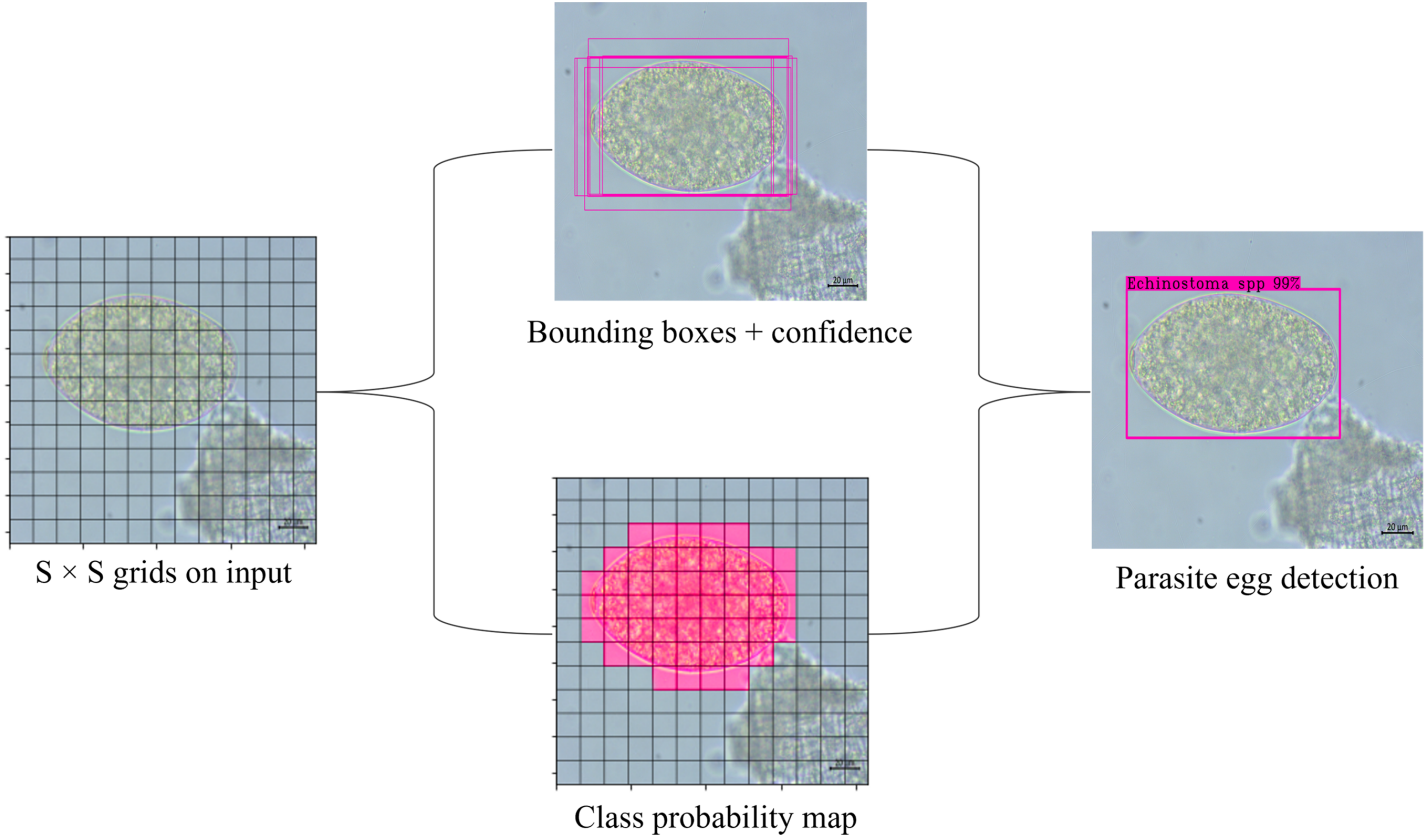
Schematic of YOLOv4-Tiny object detection algorithm for parasite egg detection.

YOLOv4-Tiny, like the other YOLO model, divides the inputted image into grids of size S × S (*S* = 13 and 26, as showed in Fig. 5) in the prediction process. For each grid, the network utilizes three anchors to recognize objects in an image. In this model, there were six pre-defined boxes of the three anchors: 10, 14, 23, 27, 37, 58, 81, 82, 135, 169, and 344, 319. The model predicts S × S ×3 bounding boxes with a confidence score and their corresponding classes if the object belongs with a grid cell. Each bounding box consists of the width (*w*), heigh (*h*), center coordinate of box (*x, y*) and object confidence (*c*). The confidence score of an object is defined as:

(1) \begin{eqnarray*}\text{Confidence Score}=\mathrm{P}(\text{object})\times Io{U}_{\text{predict}}^{\text{truth}}\end{eqnarray*}

where *P(object)* means the probability of an object. If the object is in the current grid cell, *P(object)* = 1; otherwise, 0. *IoU* stands for intersection over union value (ranging from 0 to 1) which refers to the intersection area between the predicted and ground truth bounding boxes. The threshold values and non-maximum suppression (NMS) method are used to keep the best predicted bounding box. Bounding boxes with object confidence less than the specified threshold will be removed. Finally, NMS is employed to eliminate the redundant bounding boxes. The prediction process is shown in Fig. 6.

### Parasite egg detection with other state-of-the-art object detection models

In this study, we attempted to compare model performance with two other state-of-the-art object detection models: YOLOv3 and YOLOv3-Tiny (Figs. S1 and S2). Redmon and Farhadi proposed the YOLOv3 model, the third version of YOLO (Redmon & Farhadi, 2018). The Darknet-53 backbone was used in the YOLOv3 architecture for feature extraction, and a Resnet short cut connection was added to avoid gradient disappearance. Furthermore, the YOLOv3 used a feature pyramid network for three scale feature maps, a sum squared error loss and a logistic regression function for bounding box predictions, and a logistic classifier function and a binary cross-entropy loss function for multilabel class predictions. YOLOv3 finally predicted three branches of output as shown in Fig. S1. The three feature maps of output prediction corresponded to the input images, which were 608 × 608 pixels in size, and were the resolution of 19 × 19, 38 × 38, and 76 × 76 pixels, respectively. Table 2 shows the hyperparameters used in the YOLOv3 model training.

**Table 2:** Hyper-parameters for training of YOLOv3 and YOLOv3-Tiny model.

Parameters	Values
Batch size	64
Maximum batches	500200
Subdivision	16
Momentum	0.9
Weight decay	0.005
Activation function	Leaky ReLU, Linear
Base learning rate	0.001
Step value	[400000, 450000]
Learning rate scale	[0.1, 0.1]

The simplified version of YOLOv3 is also known as YOLOv3-Tiny, and it is widely used in object detection due to the benefits of running faster and using less memory. In comparison with YOLOv3, YOLOv3-Tiny eventually produced two-branch outputs for object prediction. Figure S2 shows that images with a resolution of 416 × 416 pixels were chosen for the input layer, and feature maps with resolutions of 13 × 13 pixels and 26 × 26 pixels were used to predict output layer for objects of various sizes. Table 2 describes the hyperparameters used in the YOLOv3-Tiny model training.

### Evaluation metrics

The trained YOLO models were tested using new images of intestinal parasitic objects from the testing dataset, including protozoan cysts and helminthic eggs. The confusion matrix of the three models was used to evaluate the measuring parameters of precision, sensitivity, specificity, accuracy, and F1 score (Chu, 1999; Kittichai et al., 2021). The confusion matrix provided the following four possible interpretations of the test results: true positive, false positive, false negative, and true negative. The five measuring parameters are calculated based on these interpretations as follows:

(2) \begin{eqnarray*}\text{Precision}& & = \frac{\mathrm{TP}}{\mathrm{TP}+\mathrm{FP}} \end{eqnarray*}

(3) \begin{eqnarray*}\text{Sensitivity} \left( \mathrm{or} \right) \text{Recall}& & = \frac{\mathrm{TP}}{\mathrm{TP}+\mathrm{FN}} \end{eqnarray*}

(4) \begin{eqnarray*}\text{Specificity}& & = \frac{\mathrm{TN}}{\mathrm{TN}+\mathrm{FP}} \end{eqnarray*}

(5) \begin{eqnarray*}\text{Accuracy}& & = \frac{\mathrm{TP}+\mathrm{TN}}{\mathrm{TP}+\mathrm{FP}+\mathrm{TN}+\mathrm{FN}} \end{eqnarray*}

(6) \begin{eqnarray*}\mathrm{F}1\text{score}& & =2\times \frac{\text{Precision}\times \text{Recall}}{\text{Precision}+\text{Recall}} .\end{eqnarray*}

The evaluation metric calculations presented above were appropriate for binary classification. The one-vs-rest approach was used in multiple-label classification to calculate those metrics for each class. In the cause of imbalance class in testing datasets, micro-averaging was preferred over macro-averaging (Maslej-Krešňáková et al., 2020). The following are the micro-averaging calculations of the evaluation metrics in this study:

(7) \begin{eqnarray*}\text{Precision}& & = \frac{\sum _{\mathrm{i}=1}^{\mathrm{n}}{\mathrm{TP}}_{\mathrm{i}}}{\sum _{\mathrm{i}=1}^{\mathrm{n}}{\mathrm{TP}}_{\mathrm{i}}+{\mathrm{FP}}_{\mathrm{i}}} \end{eqnarray*}

(8) \begin{eqnarray*}\text{Sensitivity} \left( \mathrm{or} \right) \text{Recall}& & = \frac{\sum _{\mathrm{i}=1}^{\mathrm{n}}{\mathrm{TP}}_{\mathrm{i}}}{\sum _{\mathrm{i}=1}^{\mathrm{n}}{\mathrm{TP}}_{\mathrm{i}}+{\mathrm{FN}}_{\mathrm{i}}} \end{eqnarray*}

(9) \begin{eqnarray*}\text{Specificity}& & = \frac{\sum _{\mathrm{i}=1}^{\mathrm{n}}{\mathrm{TN}}_{\mathrm{i}}}{\sum _{\mathrm{i}=1}^{\mathrm{n}}{\mathrm{TN}}_{\mathrm{i}}+{\mathrm{FP}}_{\mathrm{i}}} \end{eqnarray*}

(10) \begin{eqnarray*}\text{Accuracy}& & = \frac{\sum _{\mathrm{i}=1}^{\mathrm{n}}{\mathrm{TP}}_{\mathrm{i}}+{\mathrm{TN}}_{\mathrm{i}}}{\sum _{\mathrm{i}=1}^{\mathrm{n}}{\mathrm{TP}}_{\mathrm{i}}+{\mathrm{FP}}_{\mathrm{i}}+{\mathrm{TN}}_{\mathrm{i}}+{\mathrm{FN}}_{\mathrm{i}}} \end{eqnarray*}

where *i* stands for each testing class and n is 34 classes for this study. If *i* = 1, the first class was considered positive and the remaining 33 classes negative.

To compare the models, we also used the Precision–Recall (PR) curve to evaluate the models using the area under the curve score, which measures the area under the PR curve. The PR curve was created by plotting the models’ precision and recall using the model’s confidence threshold functions. Because the PR curve is also a two-dimensional graph, Recall is plotted on the *x*-axis and Precision on the *y*-axis. The PR curve was given a model-wide evaluation, and the area under the Precision–Recall curve (AUPRC) score was effective in multilabel classification comparisons (Davis & Goadrich, 2006).

## Results

The three YOLO-based models were trained and tested on a computer running Ubuntu 16.4 LTS (64-bit) with the following specifications: Processor: Intel^®^ Core i5-8400; CPU @ 2.8 GHz*6; Memory: 31.3 GiB; and GPU: GeForce GTX 1070 Ti. Each model training took a maximum of three days to compute.

### YOLO-Based parasitic product visualization

**Figure 7:**
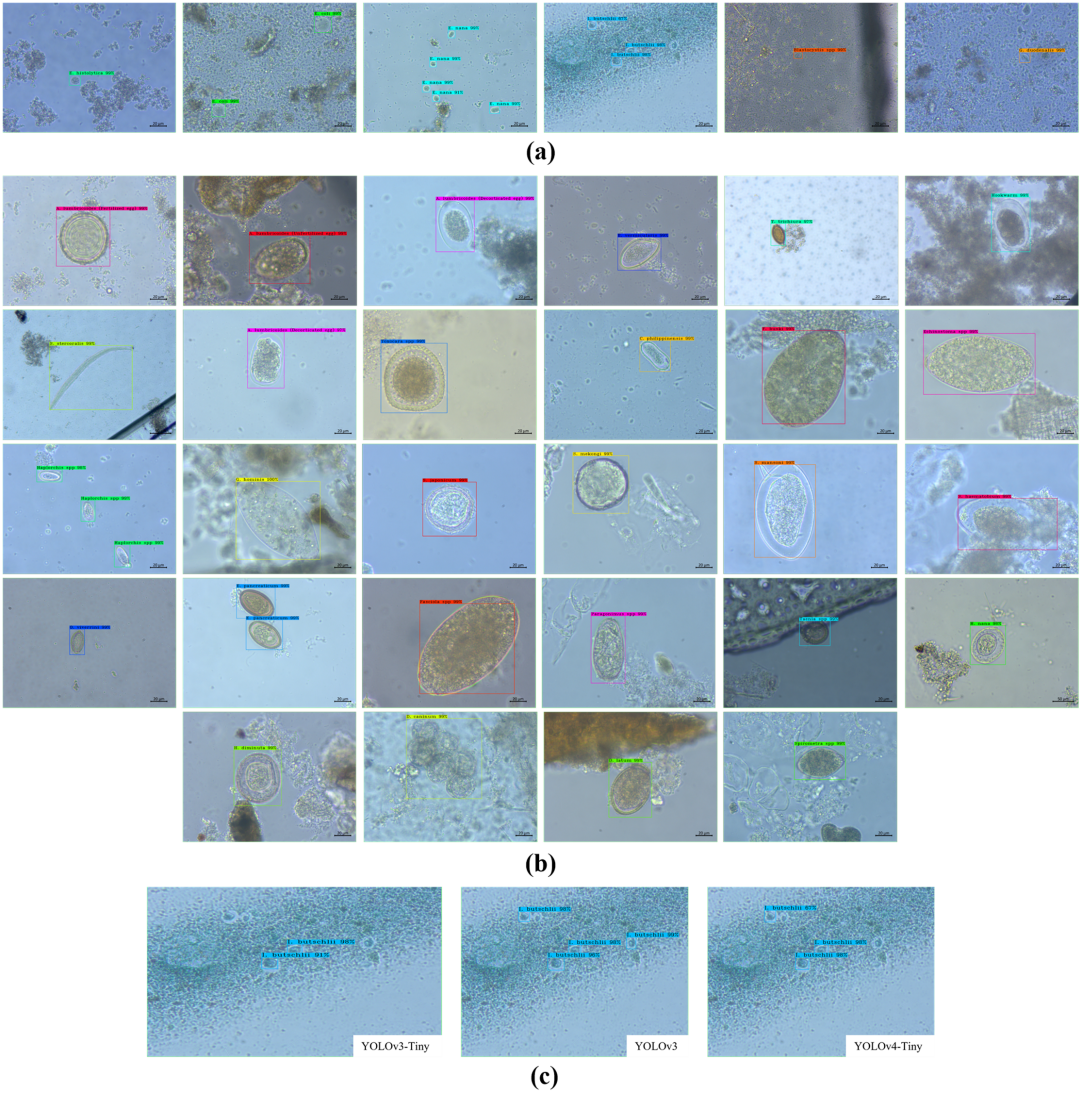
Some recognition results. (A) Protozoan cysts recognition using YOLOv4-Tiny models, (B) helminthic eggs recognition using YOLOv4-Tiny model, (C) the detection rate comparison using the three YOLO models.

**Figure 8:**
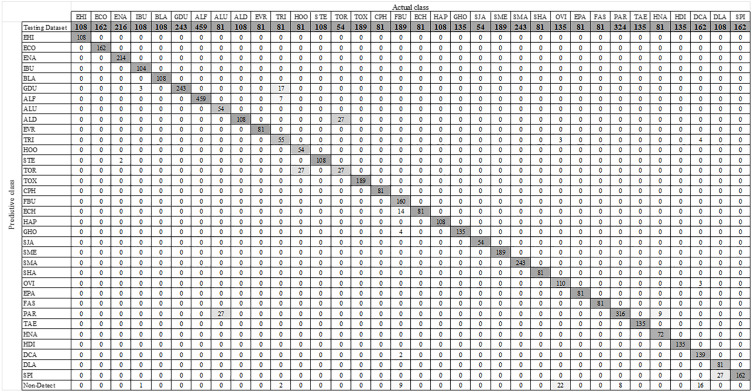
Confusion matrix for YOLOv4-Tiny model.

**Figure 9:**
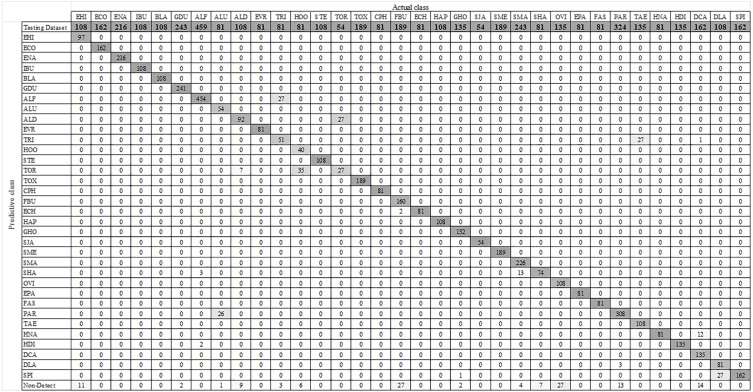
Confusion matrix for YOLOv3-Tiny model.

**Figure 10:**
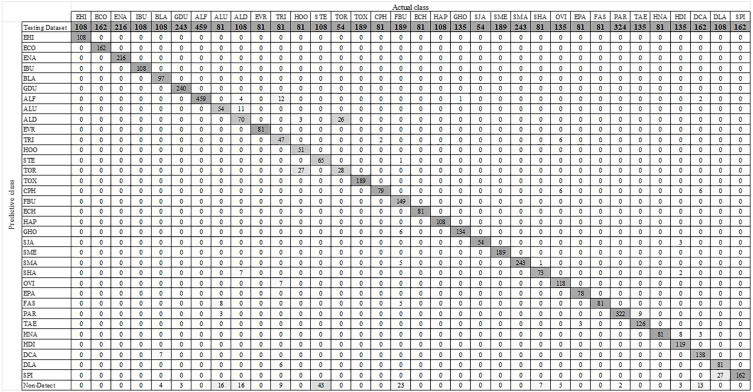
Confusion matrix for YOLOv3 model.

Figure 7 shows some YOLO-based visualization results for the localization and classification of various parasitic eggs with its detection percentages; Fig. 7A shows the recognition of protozoan cysts and Fig. 7B shows the recognition of helminthic eggs using the YOLOv4-Tiny model. In addition, the video record of recognition in the original image testing dataset (Video S1) and the video record of recognition in detecting intestinal parasitic objects, particularly *Endolimax nana*, from fecal specimens by YOLOv4-Tiny model (Video S2) are illustrated in the Supplemental Information. Because of their small size and close proximity, the three YOLO models can predict more than one parasitic egg within an image. Furthermore, even though we used the NMS value, the model produced duplicate detections for a single parasite egg. In these two cases, we count the most confident one as the output recognition result, and the sum of the counting results becomes the confusion matrix for each YOLO model, as shown in Figs. 8–10. According to the confusion matrix, YOLOv4-Tiny performs better in non-detect images than the other two YOLO models (YOLOv4-Tiny, YOLOv3-Tiny, and YOLOv3 have 58, 126, and 144 images). The detailed comparisons of the YOLOv4-Tiny model’s class-wise recognition and the three model-wise recognition results are described in the following subsections. Figure 7C shows that the number of detections in an image of the YOLOv3 model is greater than that of the YOLO-Tiny models for parasitic egg recognition. It means that YOLOv3 has a high recognition rate in detecting small parasite eggs within an image, whereas YOLOv4-Tiny has a high recognition rate when testing images. These visualization results suggest that the YOLOv4-Tiny model can be used to identify intestinal parasites such as protozoan cysts and helminthic eggs.

### Multiclass prediction with YOLOv4-Tiny model

Using the augmented testing image dataset, precision, sensitivity, and F1 score were used as evaluation parameters to assess the effectiveness of the trained YOLOv4-Tiny model. The evaluation results were analyzed based on the probability of real parasitic objects using the NMS default parameter of 0.4 and statistically significant level (threshold: 0.5). The statistical significance of each intestinal parasitic object was evaluated using a threshold of 0.5, which was an important part of the classification of various products of 34 intestinal parasitic pathogens. The confusion matrix in Fig. 8 shows the class-wise prediction quality of the trained YOLOv4-Tiny model. Table 3 shows the quantitative analysis of the pre-trained YOLOv4-Tiny based on the confusion matrix and evaluation metrics. We note that the YOLOv4-Tiny model achieves excellent agreement with the human trained examiner annotations in all protozoa cysts and 15 helminthic eggs classes because each class has above 90% in precision and sensitivity. Furthermore, the model can recognize most classes with significant characteristics with high precision and sensitivity. Only one class of helminthic eggs*, Trichostrongylus orientalis*, posed the greatest challenge to the model (precision and sensitivity scores of 50%) because the number of training and testing images was lower than for other classes with similar characteristics (*Ascaris lumbricoides* decorticated egg and hookworm). In addition, the model prediction provides the complicated recognition rate between classes with similar characteristics. We prefer the sensitivity, precision, and F1 scores in the class-wise evaluation of the YOLOv4-Tiny model because the specificity and accuracy score calculations are dependent on the true negative values.

**Table 3:** Class-wise precision, sensitivity and F1 score of the YOLOv4-Tiny model.

Class	Precision (%)	Sensitivity (%)	F1 score (%)	No. of testing images
**Protozoa**				
*E. histolytica*	100.00	100.00	100.00	108
*E. coli*	100.00	100.00	100.00	162
*E. nana*	100.00	99.07	99.53	216
*I. butschlii*	100.00	96.30	98.11	108
*Blastocystis* spp.	100.00	100.00	100.00	108
*G. duodenalis*	92.40	100.00	96.05	243
**Helminths**				
*A. lumbricoides* (Fertilized egg)	98.50	100.00	99.24	459
*A. lumbricoides* (Unfertilized egg)	100.00	66.67	80.00	81
*A. lumbricoides* (Decorticated egg)	80.00	100.00	88.89	108
*E. vermicularis*	100.00	100.00	100.00	81
*T. trichiura*	88.71	67.90	76.92	81
Hookworm	100.00	66.67	80.00	81
*S. stercoralis*	98.18	100.00	99.08	108
*T. orientalis*	50.00	50.00	50.00	54
*Toxocara* spp.	100.00	100.00	100.00	189
*C. philippinensis*	100.00	100.00	100.00	81
*F. buski*	100.00	84.66	91.69	189
*Echinostoma* spp.	85.26	100.00	92.05	81
*Haplorchis* spp.	100.00	100.00	100.00	108
*G. hominis*	97.12	100.00	98.54	135
*S. japonicum*	100.00	100.00	100.00	54
*S. mekongi*	100.00	100.00	100.00	189
*S. mansoni*	100.00	100.00	100.00	243
*S. haematobium*	100.00	100.00	100.00	81
*O. viverrini*	97.35	81.48	88.71	135
*E. pancreaticum*	100.00	100.00	100.00	81
*Fasciola* spp.	100.00	100.00	100.00	81
*Paragonimus* spp.	89.77	97.53	93.49	324
*Taenia* spp.	100.00	100.00	100.00	135
*H. nana*	100.00	88.89	94.12	81
*H. diminuta*	100.00	100.00	100.00	135
*D. caninum*	98.58	85.80	91.75	162
*D. latum*	100.00	75.00	85.71	108
*Spirometra* spp.	85.71	100.00	92.31	162

### Model-wise comparison between YOLOv4-Tiny, YOLOv3-Tiny, and YOLOv3

This section compares the performance of the three YOLO models model by model. The model’s performance is calculated using a micro-averaging operation based on the one-vs-rest method. Table 4 shows how the precision, sensitivity, specificity, accuracy, and F1 score are used in the comparison. The precision of the YOLOv4-Tiny model was 96.25%, the sensitivity was 95.08%, the specificity was 99.89%, the accuracy was 99.75%, and the F1 score was 95.66%. YOLOv4-Tiny outperforms the other two YOLO models, indicating that it is the best model for detecting various parasitic eggs in the testing dataset.

**Table 4:** Model-wise precision, sensitivity and F1 score of the three YOLO models with the threshold of 50% and NMS of 0.4 by using micro-averaging calculations.

Models	Precision	Sensitivity	Specificity	Accuracy	F1 score
YOLOv4-Tiny	96.25	95.08	99.89	99.75	95.66
YOLOv3-Tiny	95.40	92.87	99.86	99.66	94.11
YOLOv3	95.29	92.40	99.86	99.64	93.82

**Figure 11:**
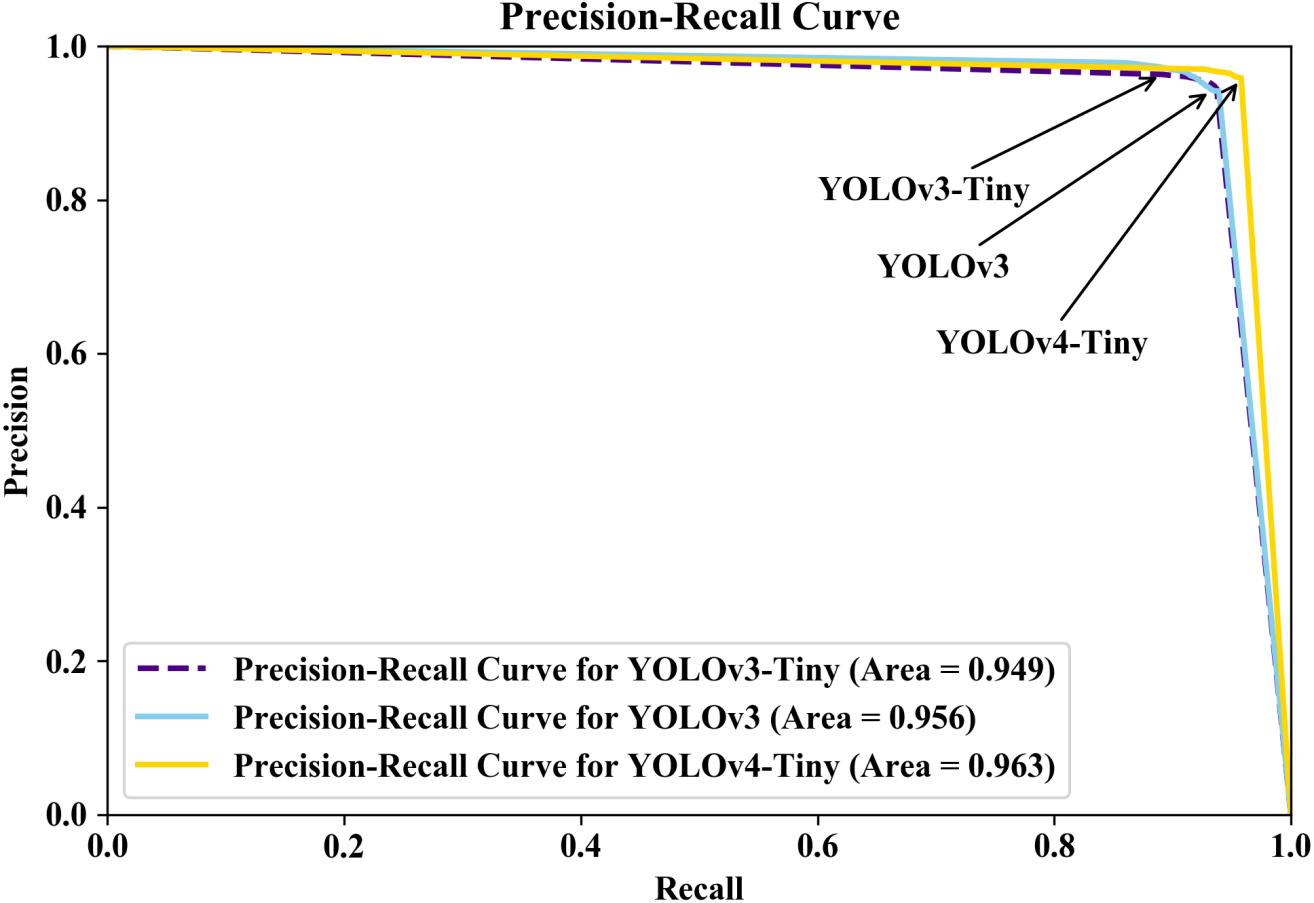
Precision-recall curve for the three models; YOLOv4-Tiny (gold color with solid line), YOLOv3-Tiny (indigo color with dashed line), YOLOv3 (sky-blue color with solid line).

Furthermore, we plotted the PR curve on the Jupyer Notebook open-source web application in a Python environment because the PR curve is a more informative and powerful plot for imbalanced dataset evaluation than the receiver operating characteristic (ROC) curve (Saito & Rehmsmeier, 2015). The PR curve, like the ROC curve, represents the trade-off between precision and recall across different thresholds with 0.05 increments (5%). Figure 11 shows the PR curves for the three YOLO models in this study. Because the AUPRC provided useful and intuitive metrics for evaluating the models’ utility (Sofaer, Hoeting & Jarnevich, 2019), we extracted the AUPRC values from the PR curve. The AUPRC was 0.963 for YOLOv4-Tiny, 0.949 for YOLOv3-Tiny, and 0.956 for YOLOv3. According to the results, the three YOLO models are outstanding models based on their performance evaluations and AUPRC values, with YOLOv4-Tiny having better ability to identify images encountered and distinguishing parasitic objects.

## Discussion

The deep learning networks rapidly developed in object detection frameworks with the continuous improvement of high-power computing machines. There are two categories in object detection approach: region proposal based two-stage detector and regression/classification based one-stage detector (Zhao et al., 2019). Indeed, the two-stage detector had high accuracy in localization and object recognition because it used RPN for object bounding boxes and RoIPool for feature extraction in candidate bounding boxes classification and regression task. The one-stage detector, on the other hand, had a high inference speed because it directly predicted the bounding boxes without the RPN step (Jiao et al., 2019). Furthermore, traditional machine learning-based digital image processing models have improved in the detection and classification of intestinal parasites. The training and testing process are the most important factors influencing the success of deep learning (Uçar et al., 2020). The training-testing ratio used in the literature ranged from 50%–95% for training and 5%–50% for testing. In our study, the YOLO approaches were designed by 90%:10% for the training-testing set for total image training and image testing, respectively (Table 1).

The intestinal parasites listed in Table 1 are mostly found in Thailand and neighboring countries in tropical and subtropical regions (Anantaphruti et al., 2007; Eom et al., 2014; Garcia, 2016; Kobayashi et al., 1996; Maipanich et al., 2004; Mori et al., 2013). In addition, several studies revealed that the prevalence of intestinal parasitic infections in Thailand remained high(Mungthin et al., 2001; Waikagul et al., 2002), and it appeared that *Ascaris lumbricoides*, *Trichuris trichiura*, and hookworm infection, including opisthorchiasis, remained major public health issues(Jiraanankul et al., 2011; Phisalprapa & Prachayakul, 2013). Another group of common intestinal protozoa worldwide were *Giardia duodenalis* and *Entamoeba histolytica*, which caused growth retardation, malabsorption, and nutritional deficiencies in the former and diarrhea and dysentery in children and liver abscess in severe cases in the latter(Matthys et al., 2011; Ngui et al., 2011). The training image datasets in this study included both protozoa and helminths.

Intestinal parasitic infections afflict people who live in unsanitary communities, have poor sanitation, and have low levels of education, particularly in remote areas (Nuchprayoon et al., 2009). This is the primary motivation for developing an effective tool to investigate local people with intestinal parasitic infections. In general, stool examination is the most basic method for detecting human intestinal parasites. Identification of each type of protozoan cyst or helminthic egg requires experience and accumulated skills on basic characteristics of each intestinal parasitic object so that pseudoparasites or contaminants in feces can be distinguished from intestinal parasitic objects. Instead of man, the YOLO approaches were expected to provide accurate results. However, there were some errors in false reporting due to contaminants that were very similar to the products of various intestinal parasites, and some reported errors involved different shapes or sizes of even the same intestinal parasites (Waikagul et al., 1997). As a result, with the design of the two-step process, the training step of YOLO models was very important; the first step was to train the YOLO models to recognize shapes, and the second was labeled images so that the YOLO models could identify and classify the intestinal parasitic objects with accurate results. Furthermore, the method of preparing fecal films from patient samples was critical because the stools contained a variety of digested food materials that passed through the digestive system, such as bubbles, oil or fat droplets, vegetable, meat, starch, and pollen grains. The appropriate fecal films prepared were neither too thick nor too thin to reduce contaminants that caused false reporting and to provide a clean background for image photographing and the construction of a set of image datasets for training. The reason for the proper amount of feces used was necessary because fecal materials could hide intestinal parasitic objects, particularly protozoan cysts, whereas too diluted fecal films with little feces were not qualified to be examined. The modified direct smear approach was developed from the following two methods: the simple direct smear (Beaver, 1949) and the modified Kato-Katz method (Katz, Chaves & Pellegrino, 1972), and it was used in this study. Both helminthic eggs and protozoan cysts were detected in the same way as in a simple direct smear, and the amount of feces used increased the chances of observing intestinal parasitic objects approximately five times with a similar known technique of modified Kato-Katz method by separating food wastes that were interrupted for searching intestinal parasitic objects and reduced fecal-contaminant background.

Following scanning, the three YOLO models automatically analyzed the images and generated a report on the presence and types of protozoan cysts or helminthic eggs, and accurate results with a precision of 95.29%–96.25% and sensitivity of 92.4%–95.08%. According to the previous report for human stool examination, two general categories of feature extraction, namely geometric characteristics and brightness description and logistic regression model, were used for recognition of four types of parasitic eggs from microscopic digital images of fecal smears, with the results reaching 99.10%–100.00% sensitivities and 98.13%–98.38% specificities (Alva et al., 2017). In our research, we looked at as many intestinal parasitic objects found in human stools as possible. As a result, each YOLO algorithm captured a series of images of protozoan cysts and helminthic eggs, which were then analyzed with an advanced algorithm capable of detecting intestinal parasitic objects such as common fecal materials and pseudoparasites (artifacts). It also detected small distractor objects or digested food such as fibers, vegetables, pollen grains, starch, fat droplets, cells, and oil droplets that affected analysis due to the impurity of the fecal samples, similar to the study by Bruun, Kapel & Carstensen (2012) with overlapping results between eggs and feces. The YOLO models were also capable of detecting the location of parasitic objects on slides. Digital image processing techniques such as noise reduction, contrast enhancement, segmentation, and other morphological processes for feature extraction and the Filtration with Steady Determinations Thresholds System for classification were used, and the result revealed that the overall rates of these detection techniques were nearly 93% in *Ascaris lumbricoides* and 94% in *Trichuris trichiura* (Ghazali, Hadi & Mohamed, 2013). Similarly, in our study, the technique of image augmentation; rotation, contrast, blur, and noise, for the expansion of new images improved the accuracy of the YOLOv4-Tiny model and increased its ability to identify images encountered and distinguish intestinal parasitic objects. A previous study addressed the 20 classes recognition of human intestinal parasite classifications using a neuro-fuzzy system with the Histogram Oriented Gradient and Linear Discriminant Analysis methods (Nkamgang et al., 2018).

The YOLO models show outstanding performance in recognizing the 34 classes of human intestinal parasitic objects in feces. To the best of the authors’ knowledge, the identification of 34 intestinal parasitic classes presented in this study is the most classes detected in the literature thus far. In addition, the class-wise prediction of the YOLOv4-Tiny shows excellent agreement with the human trained examiner annotations in 21 classes, attaining precision and sensitivity above 90%, and other classes have a moderate prediction score except for *Trichostrongylus orientalis*. Furthermore, YOLOv4-Tiny achieves 96.25% precision and 95.08% sensitivity, indicating better performance in the three YOLO-based model-wise recognition algorithms. The PR curve plotting, and YOLOv4-Tiny evaluations presented in this study also receive better accuracy, with an AUPRC of 0.963, than the other two YOLO models. The YOLOv4-Tiny model has the advantage of requiring less computing power, and its weights are around 23 megabytes in size, allowing it to train 182,058 images in approximately 3 days when using a GTX 1070 Ti GPU. According to the results, YOLOv4-Tiny is one of the best models for recognizing intestinal parasitic classes. Although the YOLOv4-Tiny model performed satisfactorily in the recognition of parasitic products in stool examination, a large number of parasitic images in image datasets were still required for the model’s improvement. Furthermore, all images used in this study were captured at a magnification of 40 × to identify the distinct features of the parasitic objects. Images at different magnifications, as well as parasite-like non-parasite material, can pose significant challenges in the recognition process. This challenge could be mitigated by the development of a better feature extractor-based object detection algorithm.

## Conclusions

Since object detection is a developing approach that has been tested for the effectiveness of detecting intestinal parasitic objects such as protozoan cysts and helminthic eggs. In comparison to the other two YOLO models, YOLOv3 and YOLOv3-Tiny, this study was carried out and proposed the YOLOv4-Tiny model for automatic recognition of a total of 34 common classes of protozoan cysts and helminthic eggs in parasitic products of stool examination. In addition, this model can be used to screen intestinal parasitic objects in stool examination as a backup option in remote areas such as Thailand’s border areas and neighboring countries where many factors supporting laboratory testing are still lacking, including enough equipment or trained personnel.

##  Supplemental Information

10.7717/peerj-cs.1065/supp-1 Supplemental Information 1 The architecture of the YOLOv3 model

10.7717/peerj-cs.1065/supp-2 Supplemental Information 2 The architecture of the YOLOv3-Tiny model

10.7717/peerj-cs.1065/supp-3 Supplemental Information 3 Video illustration of recognition in the original image testing dataset using the YOLOv4-Tiny model

10.7717/peerj-cs.1065/supp-4 Supplemental Information 4 Video record of recognition in detecting intestinal parasitic objects, particularly *Endolimax nana*, from fecal specimens by the YOLOv4-Tiny model

## Additional Information and Declarations

### Data Availability

The following information was supplied regarding data availability: The original image dataset, all trained models with weight files and detail evaluation analysis are available at Figshare: Naing, Kaung Myat; Boonsang, Siridech; Chuwongin, Santhad; Kittichai, Veerayuth; Tongloy, Teerawat; Prommongkol, Samrerng; et al. (2022): Automatic recognition of parasitic products in stool examination using object detection approach. figshare. Journal contribution https://doi.org/10.6084/m9.figshare.19200404.v3.
