# Supplementary figures and images for: Automatic recognition of parasitic products in stool examination using object detection approach
Source: PeerJ Comput Sci. 2022 Aug 17;8:e1065. doi: 10.7717/peerj-cs.1065 (PMC9455271; doi:10.7717/peerj-cs.1065)

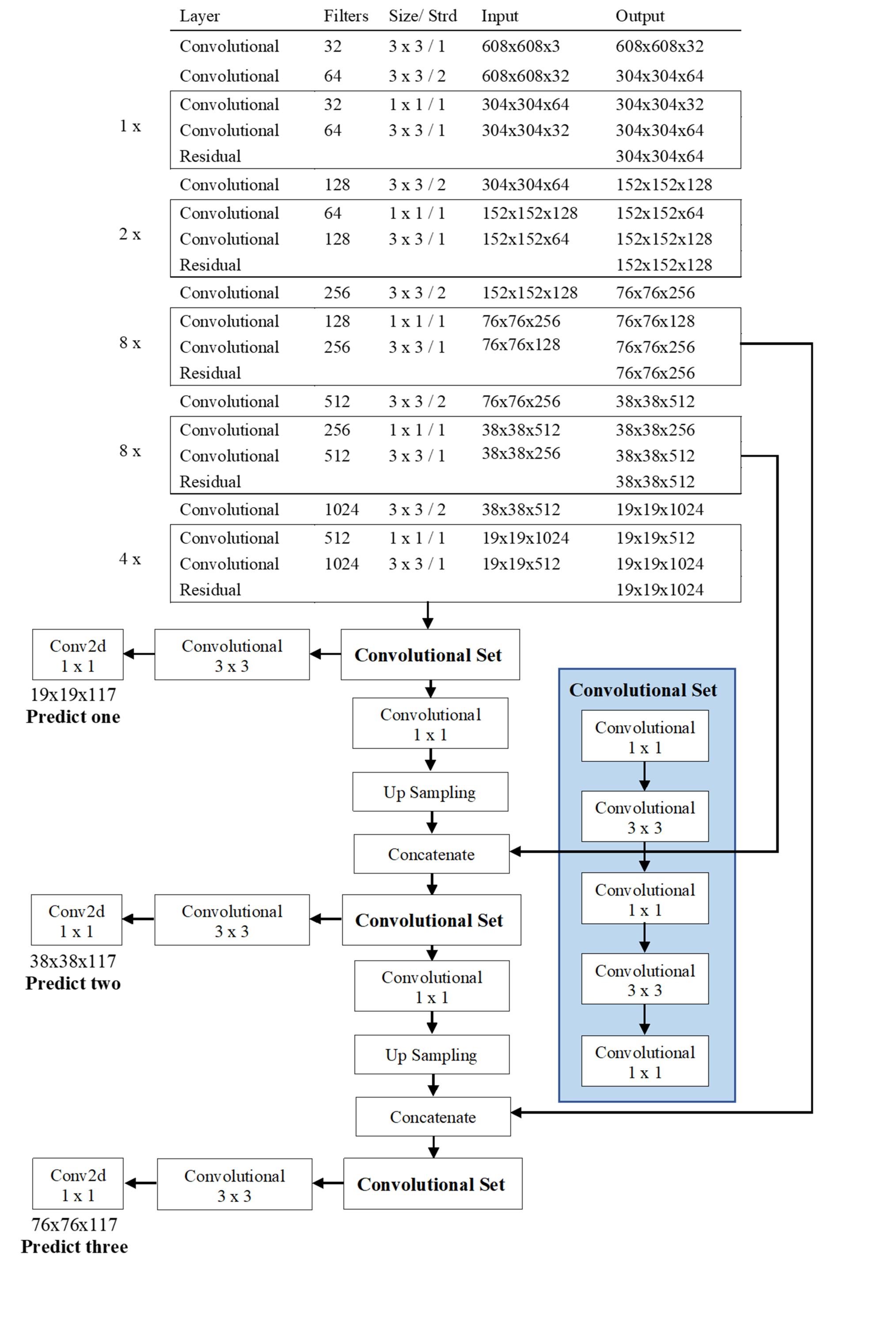

Supplement: Supplemental Information 1 [file peerj-cs-08-1065-s001.png]

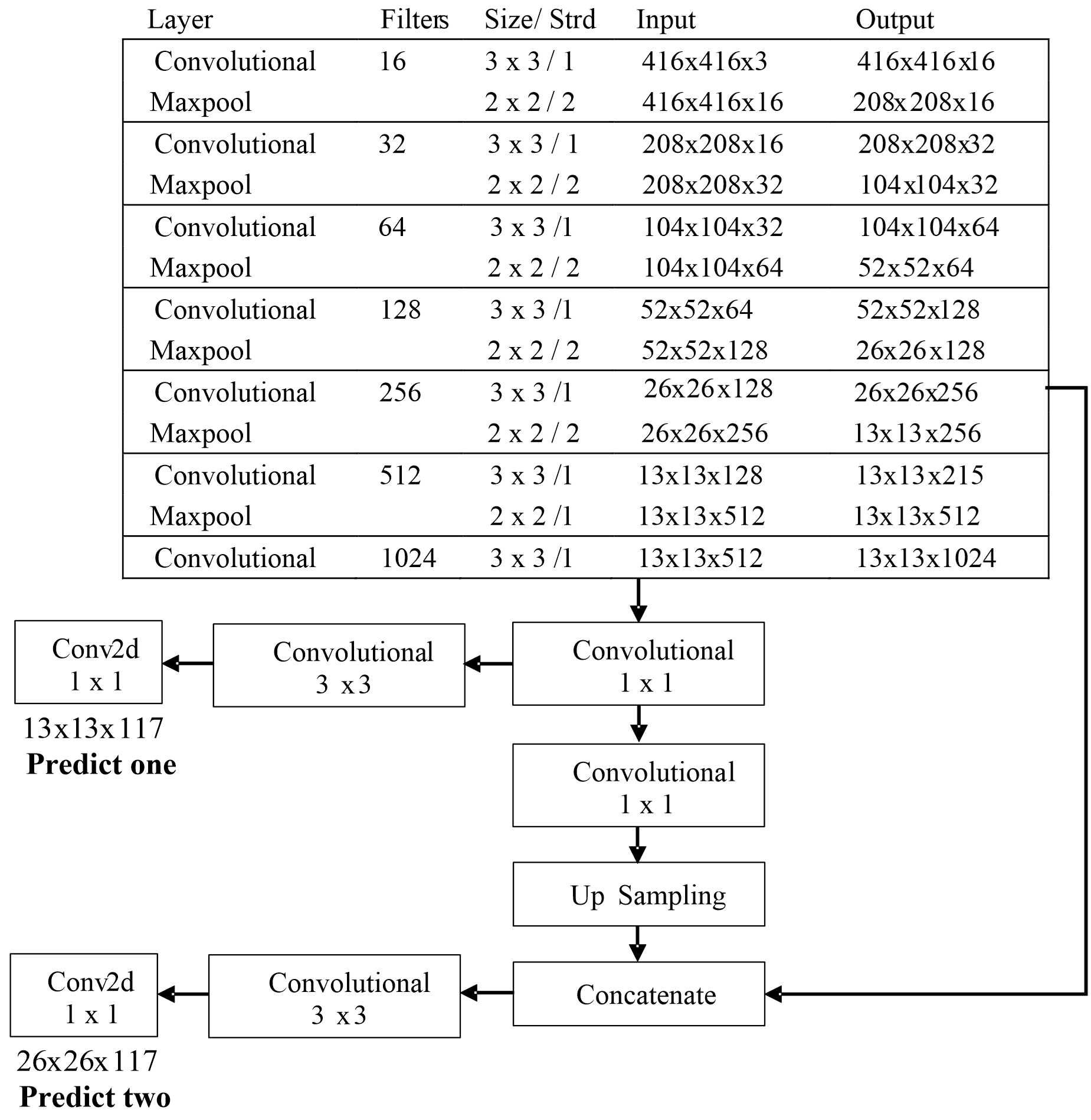

Supplement: Supplemental Information 2 [file peerj-cs-08-1065-s002.png]
